# Optimization of a Digestion Method to Determine Total Mercury in Fish Tissue by Cold Vapor Atomic Fluorescence Spectrophotometry

**DOI:** 10.3390/mps3020045

**Published:** 2020-06-23

**Authors:** Gabriela S. Yánez-Jácome, David Romero-Estévez, Hugo Navarrete, Karina Simbaña-Farinango, Pamela Y Vélez-Terreros

**Affiliations:** Centro de Estudios Aplicados en Química, Pontificia Universidad Católica del Ecuador, Quito 17012184, Ecuador; dfromero@puce.edu.ec (D.R.-E.); kjsimbanaf@puce.edu.ec (K.S.-F.); pyvelez@puce.edu.ec (P.Y.V.-T.)

**Keywords:** fish muscle, high-pressure vessels, microwave-assisted acid digestion, tilapia (*Oreochromis niloticus*)

## Abstract

Several microwave-assisted digestion methods were tested at the Centro de Estudios Aplicados en Química laboratory in Quito, Ecuador, to determine the accuracy and performance efficiency of the mineralization process for the determination of total mercury in fish tissue by cold vapor atomic fluorescence spectrophotometry. The use of MARSEasyPrep high-pressure vessels, low amounts of reagents (1 cm^3^ HNO_3_, 1 cm^3^ H_2_O_2_, and 1 cm^3^ HClO_4_), an irradiation temperature of 210 °C, and 35 min of mineralization time resulted in accurate performance, with recoveries of certified reference material DORM-4 between 90.1% and 105.8%. This is better than the Association of Official Analytical Chemists 2015.01 method, which has a reported accuracy of 81%. The repeatability precision and intermediate precision were established at three concentration levels (0.167, 0.500, and 0.833 mg·kg^−1^) and expressed as the percentage of the relative standard deviation ranging from 1.5% to 3.0% and 1.7% to 4.2%, respectively. Further, the method was satisfactorily applied to analyze fortified samples of tilapia (*Oreochromis niloticus*), with recoveries ranging from 98.3% to 104.3%. The instrumental limits of detection and quantification were 0.118 µg·dm^−3^ and 0.394 µg·dm^−3^, respectively.

## 1. Introduction

Mercury (Hg) is a ubiquitous, persistent, toxic trace metal that can be globally transported in the atmosphere [1,2]. Hg is naturally found in the Earth’s crust and is primarily released into the environment by the weathering of rocks and soils as well as volcanic activities [3]. However, anthropogenic activities, such as artisanal and small-scale gold mining, biomass burning, cement production, and chloralkali production, among others, have increased its presence in the environment [4]. Estimates of direct mercury emissions into the atmosphere from anthropogenic activities range between 2200 and 4000 megagrams per year, of which approximately 60% comes from the combustion of fossil fuels, such as coal [5,6,7]. 

Mercury is present in the environment in three chemical forms: (1) elemental mercury (Hg^0^), (2) inorganic mercury (Hg^+2^), and (3) organic mercury, principally methylmercury (MeHg) [8]. Atmospheric deposition of inorganic mercury into aquatic environments poses a serious threat because of its complex cycle, during which the chemical forms can be easily transported into water and sediment [9]. In coastal aquatic and marine sediment, mercury can be present as Hg^+2^, which reacts in anaerobic conditions through sulfate-reducing bacteria [10,11,12] to form MeHg [13]. MeHg is the most toxic form of Hg, and it can alter neurological functions [14] and affect reproductive organs, kidneys, and lungs [15]. In addition, MeHg produces effects such as teratogenicity, nephrotoxicity, and immunotoxicity [16]. This neurotoxin can penetrate the placenta and concentrate in the fetus [17], causing adverse effects on mental development/behavior and birthweight and inducing preterm delivery [18].

Methylmercury is incorporated into fish enzymes and proteins through the bond with sulphydryl groups [19], leading to its bioaccumulation in tissues and biomagnification through the aquatic food chain [20,21,22], reaching its highest concentrations in older, larger predatory fish [23,24,25]. Some studies have reported that MeHg in fish represents 64% to 100% [26] and 75% to 98% of total mercury (THg) content depending on the size and age of the fish [27]. Nevertheless, because most mercury in fish tissue is primarily present as MeHg, the US Environmental Protection Agency (EPA) recommends that THg may be determined as an approach to human health risk assessment and as a cost-effective technique [28].

Determination of trace metals, such as THg, in fish using spectroanalytic techniques requires a sample preparation step [29], such as dry ashing, wet ashing, microwave-assisted digestion, ultrasonic extraction, or slurry sample preparation [30]. Microwave-assisted digestion in closed Teflon vessels is a simple, fast dissolution technique through which organic matter is completely destroyed, minimizing reagent volume consumption, the risk of sample contamination, and loss of volatile elements [30,31]. Techniques such as thermal decomposition atomic absorption spectrometry (TD-AAS) have also been reported, where the analyses of solid and liquid samples do not require any pretreatment [32], but there are limitations due to the relatively short linear working range of AAS [30].

Different techniques are employed in the determination of THg in foodstuff samples, and the most used techniques are cold vapor atomic absorption spectrometry (CV-AAS) [26,33,34,35,36], inductively coupled plasma optical emission spectrometry (ICP-OES) [37], inductively coupled plasma mass spectrometry (ICP-MS) [38], cold vapor atomic fluorescence spectrometry (CV-AFS) [19,29,39,40], and thermal decomposition atomic absorption spectrometry (TD-AAS) [24,41,42].

The aim of this study was the development of a reliable and high-efficiency mineralization method for fish samples using microwave-assisted digestion, followed by the determination of total mercury content using spectroanalytic techniques such as CV-AFS. Reliable and suitable protocols are needed for the determination of THg in different matrixes, even though international protocols have also been developed.

## 2. Materials and Methods

### 2.1. Instrumentation

All measurements were carried out using Mercur Plus, a cold vapor analyzer based on atomic fluorescence (Analytik Jena, Jena, Germany). A low-pressure mercury lamp UVU5 was used as a radiation source. Measurements were carried out with an excitation and fluorescence wavelength of 253.7 nm. 

### 2.2. Chemical and Reagents

All solutions were prepared in high-quality reagent water (resistivity 18.2 MΩ·cm at 25 °C) obtained from a Genie 5 Direct-Pure Water System (Rephile, Shanghai, China). All chemicals used were of analytical grade. The Mercur Plus equipment works with a 2% (*w*/*v*) solution of tin (II) chloride dihydrate (Sigma Aldrich, Steinheim, Germany, Certified ACS, CAS# 10025-69-1, PubChem CID: 24479) as a reducing agent, prepared in a 4% (*v*/*v*) hydrochloric acid (Fisher Chemical, Otawwa, Canada, Certified ACS, CAS# 7647-01-0, PubChem CID: 313) solution. A 2% (*v*/*v*) hydrochloric acid solution was used as the blank, diluent, and acid solution for the Mercur Plus operation. Argon 99.999% (Linde, Quito, Ecuador, CAS# 7440-37-1, PubChem CID: 23968) was used as the carrier and purge gas.

A standard solution of 50 μg·dm^−3^ was prepared with appropriate dilution of the certified stock solution of mercury (9.995 ± 0.056 μg·cm^−3^) (Inorganic Ventures, Virginia, United States).

The cleaning procedure for all the glassware consisted of washing with soap (Green Solutions glass cleaner, pH = 7.5–8.5) and rinsing with tap water. This step was repeated twice, and the glassware was then left to soak in soap overnight. The next day, the glassware was rinsed with tap water, rinsed twice with high-quality reagent water, and left to dry upside down. After the overnight soaking in soap, the glassware used in the preparation of the standard solutions was rinsed with high-quality reagent water and soaked in 5% (*v*/*v*) nitric acid (Fisher Chemical, Otawwa, Canada, Certified ACS, CAS# CAS 7697-37-2, PubChem CID: 944) overnight, then rinsed twice with high-quality reagent water and left to dry upside down.

### 2.3. Methodology

Several acid digestion methods for fish muscle were performed using a CEM MARS 6 microwave. All digestion tests were done using the certified reference material (CRM) DORM-4 (fish protein). A 0.3 g portion was weighed directly in a polytetrafluoroethylene vessel, where the digestion took place.

After digestion, samples were cooled, filtered, and transferred to a 50 cm^3^ volumetric flask. One cubic centimeter of hydrochloric acid (Fisher Chemical, Otawwa, Canada, Certified ACS, CAS# 7647-01-0, PubChem CID: 313) was added to acidify the sample and reach the same working conditions of the equipment, i.e., HCl 2% (*v*/*v*). The volume was calibrated with high-quality reagent water.

The initial digestion tests were carried out using modified methods proposed in previous studies and are shown in Table 1. These modifications were applied after considering their adaptability to existing conditions at the Centro de Estudios Aplicados en Química (CESAQ-PUCE) laboratory as well as the availability of reagents and technical capabilities of the available equipment.

Because low recovery results were obtained when using the original methods shown in Table 1, additional tests were performed by adding different amounts of reagents and varying the technical conditions of the microwave digestion method, including irradiation temperature, time ramp, holding time, power, and pressure (Table 2).

### 2.4. Analytical Quality Assurance 

The quantification with the CV-AFS technique used multipoint calibration curves at 1, 2, 3, 4, and 5 µg·dm^−3^, which were diluted by the autosampler using the standard solution of 50 μg·dm^−3^. The calibration curve was calculated and displayed with a 95% confidence level, considering the linear regression coefficients (R^2^ > 0.99).

The instrumental limit of detection (LOD) and limit of quantification (LOQ) were determined on the variability of digested blanks. LOD and LOQ were calculated by multiplying the standard deviation of the mean blank concentration values by 3 and 10, respectively. The LOD was 0.118 µg·dm^−3^, and the LOQ was 0.394 µg·dm^−3^.

The accuracy of the methodology was established by analyzing CRM DORM-4, with THg recovery calculated according to the certified value of 412 ± 36 µg·kg^−1^.

## 3. Results and Discussion

The Association of Official Analytical Chemists (AOAC) Official Method 2015.01 for Heavy Metals in Food [43] was first performed using Xpress digestion vessels and the CEM MARS 6 microwave digestion system. This method suggests the addition of 0.1 cm^3^ of a 50 mg·dm^−3^ solution of gold (Au) to stabilize Hg in the preparation and lutetium (Lu) to assess the potential loss of the analyte during the microwave digestion process. However, this methodology was modified because a solution of Au and Lu was not available at CESAQ-PUCE. When this method was tested, a recovery of 54.6% was achieved.

Results obtained using Adel et al.’s method [44] showed that digestion with open systems, such as hot plate digestion, had low mercury recovery; this could be due to the loss of the analyte by volatilization. Nevertheless, the open system proposed by Horvat et al. [47] resulted in a recovery of 90.3%, in which the use of strong acids such as H_2_SO_4_ and HNO_3_ allows the complete mineralization of organic matter [49]. However, this method requires one hour at room temperature to react and releases gases during the digestion, making it necessary to work inside a fume hood. 

The same preparation method proposed by Horvat et al. [47] was performed in MARSXpress vessels with microwave-assisted digestion, considering that microwave heating in closed vessels guarantees temperature feedback control with extremely low contamination risk [31]; however, the recovery of the CRM was 69.3% (Table 2). The digestion step proposed by Qin et al. [45] was also performed, but the 100% power of the MARS 6 microwave, the equipment available at CESAQ-PUCE, corresponded to 1800 W, which differed from the 1600 W used by the authors. The recovery result obtained was 60.2%, meaning more digestion methods had to be tested, assuming that the methods performed did not allow complete mineralization of the sample or that Hg volatilization might have occurred during the process.

The digestion method proposed by Herrero Fernández et al. [46] was also conducted in the MARS 6 microwave, in which 8 cm^3^ of HNO_3_ and 2 cm^3^ of H_2_O_2_ (Fisher Chemical, Otawwa, Canada, Certified ACS, CAS# 7722-84-1, PubChem CID: 784) were used. The CRM recovery was 66.7%; however, the equipment employed might have had different technical capabilities in comparison to the ETHOS 1 Milestone microwave used by Herrero Fernández et al. [46]. Furthermore, the volume of HNO_3_ and H_2_O_2_ might be the most important parameter to take into account to develop a reliable digestion method.

When using an irradiation temperature of 130 °C, recommended by Hight and Cheng [31], the CRM recovery was 49.3%; however, higher temperatures were used during the current study. This demonstrates that using a higher temperature does not compromise the accuracy of the results or lead to losses of volatile species during digestion, as commented by Shah et al. [48]; thus, lower temperatures are not required [36].

The use of MARSXpress vessels with different reagent amounts and several microwave digestion programs (Table 2) showed low mercury recoveries ranging between 52.6% and 71.0%. Therefore, utilizing high-pressure decomposition vessels such as MARSEasyPrep instead of varying the irradiation temperature could be necessary.

The same digestion method proposed by Qin et al. [45] was performed using MARSEasyPrep vessels with the addition of 1 cm^3^ HClO_4_, and 77.1% CRM recovery was achieved. This shows that even though microwave-assisted digestion in closed flasks reduces the risk of loss and is a highly efficient decomposition technique [32], the mineralization may be incomplete depending on the pressure, temperature, heating time, and amount of reagents added [50].

Shah et al. [48] proposed the addition of 2 cm^3^ of a freshly prepared mixture of concentrated HNO_3_ and H_2_O_2_ (1:1, *v*/*v*) and a microwave digestion method with a heating program for 2–3 min at 80% total power. This method was applied in the CESAQ-PUCE laboratory, considering that the MARS 6’s capabilities do not permit extremely short irradiation times, with a minimum ramp time of 20 min. CRM recovery was 65.2%.

### 3.1. Optimization of the Microwave-Assisted Digestion Method

Despite all the microwave conditions tested and the different amounts of reagents added, the method that showed results within the certified value (with recoveries between 90.1% and 105.8%) corresponded to the use of high-pressure vessels, such as MARSEasyPrep, low amounts of reagents (1 cm^3^ HNO_3_, 1 cm^3^ H_2_O_2_, and 1 cm^3^ HClO_4_), gentle mixing of the samples, and leaving the vials open for 10 min before closing them. In the present study, the addition of HClO_4_ (Fisher Chemical, Otawwa, Canada, PubChem CID:24247) is suggested to achieve a complete digestion of organic matter, which will not be reached with the relatively low oxidation potential of nitric acid and hydrogen peroxide [30]. This was verified with Shah et al.’s [48] proposed method and with that developed by CESAQ-PUCE.

The accuracy of the developed method is higher (95.2%) than that reported by the AOAC Official Method 2015.01 (81.0%) in the same CRM DORM-4. Therefore, there is no need to add Au solutions or NaCl to digestion vessels to stabilize Hg and prevent its loss [31,43].

The optimized microwave digestion program is shown in Table 3.

### 3.2. Analytical Features of the Proposed Method

The method’s performance was evaluated in terms of repeatability and intermediate precision. For repeatability, triplicates of fortification analyses at three levels (0.167, 0.500, and 0.833 mg·kg^−1^) were carried out each day. For intermediate precision, groups of the triplicate trials were carried out during five different nonconsecutive days. Results were evaluated by calculating the relative standard deviation (RSD, %). All the R^2^ values were higher than 0.9955, showing the linear adjustment of the calibration curves. 

The percentage of combined and expanded uncertainties were estimated as 9.05% and 18.1%, respectively. 

### 3.3. Applications

The digestion method that was developed was applied to determine the amount of THg in tilapia (*Oreochromis niloticus*) samples. Tilapia is a widely cultivated, commercial omnivorous freshwater fish, which was chosen for its low THg concentration with a mean value of 7.5µg·kg^−1^ (wet weight). Moisture content in fish muscle was determined using a Moisture Analyzer HB43-S (Mettler Toledo, Greifensee, Switzerland) with a mean value of 77.3%. Samples were dried to a constant weight in a Memmert UM 500 stove (Memmert, Schwabach, Germany) and subsequently powdered using a ball mill MM 400 (Retsch, Retsch, Germany).

A recovery study was performed in triplicate at three different concentration levels by fortifying fish muscle samples. The results are shown in Table 4. Recovery values were 104.3%, 99.5%, and 98.3% for each fortification level. The RSD values for the intermediate precision were 4.2%, 1.7%, and 3.4% at the lowest, medium, and highest concentrations assayed, respectively.

## 4. Conclusions

The mineralization process developed in this study is suitable for determining the amount of THg content through CV-AFS in fish samples, providing the following advantages: (1) small reagent volumes, (2) short digestion time, (3) complete digestion of the sample, leading to a clear solution with no solids remaining, and (4) low risk of loss through volatilization of the analyte. Furthermore, glassware cleaning is quite simple, with no need for several washing steps and high amounts of reagents, such as nitric acid.

According to the results, the novel method has better accuracy and performance efficiency than the international protocol tested, i.e., the AOAC 2015.01 method.

The results obtained in this study also shows the usefulness of MARSEasyPrep vessels in sample treatment, with higher pressures and the use of perchloric acid allowing complete mineralization and relatively high sample throughput.

## Figures and Tables

**Table 1:** Technical characteristics of reference procedures for digestion of fish samples to determine total mercury.

Original Features of the References										Modifications
Digestion Technique	Sample Amount (g)	Reagents for Digestion	Temperature (°C)	Ramp Time (min)	Holding Time (min)	Power (W)	Pressure (psi)	Recovery Obtained (%)	Reference	
Microwave-assisted digestion	0.25	4 cm^3^ HNO_3_ 1 cm^3^ H_2_O_2_ 0.1 cm^3^ of 50 mg·dm^−3^ Au + Lu solution	190	20	10	No power value described	No pressure value described	54.5	[43]	0.3 g of sample amount was used. No addition of 0.1 cm^3^ of the 50 mg·dm^−3^ Au + Lu solution to each digestion vessel. Power set to 1440 W. Pressure not applied. MARSXpress vessels were used.
Wet ashing, hot plate	0.3	5 cm^3^ HNO_3_ 4 cm^3^ H_2_O_2_ 1 cm^3^ HClO_4_	200	---	---	---	---	55.7	[44]	0.3 g of sample amount was used. No addition of 45 mg of V_2_O_5_ to samples and no dilution to 50 cm^3^ with 20 cm^3^ distilled water and K_2_Cr_2_O_7_ (% 2). Pressure not applied.
Microwave-assisted digestion	0.5 g 1.0 g 2.0 g	1.0 ± 0.01 cm^3^ NaCl 1% (*w*/*v*) 5.0 ± 0.1 cm^3^ HNO_3_	130	10	---	300	No pressure value described	49.3	[31]	0.3 g of sample amount was used. Ramp temperature was modified from 5 to 10 min for heating from ambient to 130 °C. No dilution to 50 cm^3^ adding 3.5 ± 0.1 cm^3^ HCl. Pressure not applied. MARSXpress vessels were used.
				---	20	1200				
Microwave-assisted digestion	0.5	5 cm^3^ HNO_3_ 2 cm^3^ H_2_O_2_	110	5	3	1600	No pressure value described	60.2	[45]	0.3 g fish muscle was used. Power at 100% for the equipment used by the reference authors was 1600 W; we used 1800 W as per the CEM MARS 6 specifications. Pressure not applied. MARSXpress vessels were used.
			150	4	8	1600				
			180	3	25	1600				
Microwave-assisted digestion	0.2	8 cm^3^ HNO_3_ 2 cm^3^ H_2_O_2_	180	20	50	No power value described	No pressure value described	66.7	[46]	0.3 g fish muscle was used. Power set at 1600 W. Pressure not applied. MARSXpress vessels were used.
Wet ashing, hot plate	0.5	3 cm^3^ HNO_3_ 1 cm^3^ HClO_4_ 5 cm^3^ H_2_SO_4_	230	---	---	---	---	90.3	[47]	Method not modified at CESAQ-PUCE.
Microwave-assisted digestion	0.5	5 cm^3^ HNO_3_ 2 cm^3^ H_2_O_2_	110	15	3	1600	No pressure value described	77.1	[45]	0.3 g fish muscle was used. Addition of 1 cm^3^ of HClO_4_ to each vessel. Pressure set at 800 psi. Power at 100% for the equipment used by the reference authors was 1600 W; we used 1800 W as per the CEM MARS 6 specifications. MARSEasyPrep vessels were used.
			150	4	8	1600				
			180	3	25	1600				
Microwave-assisted digestion	0.2	1 cm^3^ HNO_3_ 1 cm^3^ H_2_O_2_	No temperature value described	Microwave heating program for 2–3 min.		80% of total power (900 W)	No pressure value described	65.2	[48]	0.3 g fish muscle was used. Power and pressure set at 1400 W and 800 psi, respectively. Temperature set at 210 °C, ramp time: 20 min, holding time: 15 min. No dilution to 25 cm^3^ of volumetric flask with 0.1 M HCl. MARSEasyPrep vessels were used.

**Table 2:** Technical characteristics of microwave digestion methods tested for fish samples to determine total mercury.

Type of Vessel	Sample Amount (g)	Reagents for Digestion	Temperature (°C)	Ramp Time (min)	Holding Time (min)	Power (W)	Pressure (psi)	Recovery (%)
MARSXpress	0.3	4 cm^3^ HNO_3_ 1 cm^3^ H_2_O_2_	190	20	10	1440	no pressure control used	70.5
MARSXpress	0.3	5 cm^3^ HNO_3_ 4 cm^3^ H_2_O_2_ 1 cm^3^ HClO_4_	200	20	10	1440	no pressure control used	68.3
MARSXpress	0.3	5 cm^3^ HNO_3_ 4 cm^3^ H_2_O_2_ 1 cm^3^ HClO_4_	180	10	15	1800	no pressure control used	62.9
MARSXpress	0.3	5 cm^3^ HNO_3_ 4 cm^3^ H_2_O_2_ 1 cm^3^ HClO_4_	180	25	15	1800	no pressure control used	64.3
MARSXpress	0.3	5 cm^3^ HNO_3_ 4 cm^3^ H_2_O_2_ 1 cm^3^ HClO_4_	180	25	30	1800	no pressure control used	66.5
MARSXpress	0.3	5 cm^3^ HNO_3_ 4 cm^3^ H_2_O_2_ 1 cm^3^ HClO_4_	200	25	20	1800	no pressure control used	67.9
MARSXpress	0.3	5 cm^3^ HNO_3_ 4 cm^3^ H_2_O_2_ 1 cm^3^ HClO_4_	190	25	20	1800	no pressure control used	71.0
MARSXpress	0.3	5 cm^3^ HNO_3_ 4 cm^3^ H_2_O_2_ 1 cm^3^ HClO_4_	200	25	25	1200	no pressure control used	65.2
MARSXpress	0.3	5 cm^3^ HNO_3_ 4 cm^3^ H_2_O_2_ 1 cm^3^ HClO_4_	200	25	25	1400	no pressure control used	61.0
MARSXpress	0.3	5 cm^3^ HNO_3_ 4 cm^3^ H_2_O_2_ 1 cm^3^ HClO_4_	130	10		300	no pressure control used	52.6
					20	600		
MARSXpress	0.3	1 cm^3^ HNO_3_ 1 cm^3^ HClO_4_ 5 cm^3^ H_2_SO_4_	200	20	35	1800	no pressure control used	69.3
MARSEasyPrep	0.3	5 cm^3^ HNO_3_ 4 cm^3^ H_2_O_2_ 1 cm^3^ HClO_4_	200	15	15	1400	800	79.8
MARSEasyPrep	0.3	5 cm^3^ HNO_3_ 4 cm^3^ H_2_O_2_ 1 cm^3^ HClO_4_	200	25	25	1400	800	73.5
MARSEasyPrep	0.3	5 cm^3^ HNO_3_ 4 cm^3^ H_2_O_2_ 1 cm^3^ HClO_4_	200	25	25	1400	800	84.1
MARSEasyPrep	0.3	1 cm^3^ HNO_3_ 1 cm^3^ H_2_O_2_ 1 cm^3^ HClO_4_	200	15	15	1400	800	86.9
MARSEasyPrep	0.3	1 cm^3^ HNO_3_ 1 cm^3^ H_2_O_2_ 1 cm^3^ HClO_4_	220	20	15	1400	800	94.4
MARSEasyPrep	0.3	1 cm^3^ HNO_3_ 1 cm^3^ H_2_O_2_ 1 cm^3^ HClO_4_	210	20	15	1400	800	95.2

**Table 3:** Microwave digestion program.

Step	Temperature (°C)	Ramp Time (min)	Holding Time (min)	Power (watts)	Pressure (psi)
1	210	20	15	1400	800

**Table 4:** Recovery of total mercury in fortifications of fish muscle samples.

Concentration Expected (mg·kg^−1^)	Concentration Found ^a^ (mg·kg^−1^)	Highest Repeatability Precision RSD ^a^ (%)	Intermediate Precision RSD ^a^ (%)	Recovery (%)
0.167	0.174	3.0	4.2	104.3
0.500	0.498	1.5	1.7	99.5
0.833	0.819	2.7	3.4	98.3

^a^ Mean (n = 15): Triplicates of fortification analyses during five different days.
